# Riparian leaf litter decomposition on pond bottom after a retention on floating vegetation

**DOI:** 10.1002/ece3.5488

**Published:** 2019-07-31

**Authors:** Ya‐Lin Zhang, Wei‐Jun Zhang, Jun‐Peng Duan, Xu Pan, Guo‐Fang Liu, Yu‐Kun Hu, Wen‐Bing Li, Yue‐Ping Jiang, Jian Liu, Wen‐Hong Dai, Yao‐Bin Song, Ming Dong

**Affiliations:** ^1^ Key Laboratory of Hangzhou City for Ecosystem Protection and Restoration, College of Life and Environmental Sciences Hangzhou Normal University Hangzhou China; ^2^ State Key Laboratory of Vegetation and Environmental Change, Institute of Botany Chinese Academy of Sciences Beijing China; ^3^ Institute of Wetland Research Chinese Academy of Forestry Beijing China; ^4^ Hangzhou Xixi National Wetland Park Research Centre for Ecological Sciences Hangzhou China; ^5^ Institute of Environmental Research Shandong University Qingdao China

**Keywords:** floating vegetation, leaf litter decomposition, mass loss, nutrient loss, retention

## Abstract

Allochthonous (e.g., riparian) plant litter is among the organic matter resources that are important for wetland ecosystems. A compact canopy of free‐floating vegetation on the water surface may allow for riparian litter to remain on it for a period of time before sinking to the bottom. Thus, we hypothesized that canopy of free‐floating vegetation may slow decomposition processes in wetlands. To test the hypothesis that the retention of riparian leaf litter on the free‐floating vegetation in wetlands affects their subsequent decomposition on the bottom of wetlands, a 50‐day in situ decomposition experiment was performed in a wetland pond in subtropical China, in which litter bags of single species with fine (0.5 mm) or coarse (2.0 mm) mesh sizes were placed on free‐floating vegetation (dominated by *Eichhornia crassipes*, *Lemna minor*, and *Salvinia molesta*) for 25 days and then moved to the pond bottom for another 25 days or remained on the pond bottom for 50 days. The leaf litter was collected from three riparian species, that is, *Cinnamomum camphora*, *Diospyros kaki*, and *Phyllostachys propinqua*. The retention of riparian leaf litter on free‐floating vegetation had significant negative effect on the carbon loss, marginal negative effects on the mass loss, and no effect on the nitrogen loss from leaf litter, partially supporting the hypothesis. Similarly, the mass and carbon losses from leaf litter decomposing on the pond bottom for the first 25 days of the experiment were greater than those from the litter decomposing on free‐floating vegetation. Our results highlight that in wetlands, free‐floating vegetation could play a vital role in litter decomposition, which is linked to the regulation of nutrient cycling in ecosystems.

## 1 INTRODUCTION

Allochthonous litter input has been largely reported to be a major source of organic carbon that supports food webs and thus influences the trophic structures of ecosystems (Pintar & Resetarits, 2017; Rubbo, Cole, & Kiesecker, 2006; Stoler & Relyea, 2011, 2016). Local soil and/or water conditions have generally been considered among the main factors that affect the decomposition of plant litter, assuming that litter reaches the ground immediately and directly after senescence or death (Berg & McClaugherty, 2014; Graça et al., 2015; Lavelle, Blanchart, Martin, Martin, & Spain, 1993). However, due to the vertical stratification of the vegetation canopy in some ecosystems, some litter could remain on the canopy for a period of time before landing on the ground where decomposition usually and mainly happens. In this case, such retention will lead to dramatic differences in abiotic and biotic environments, and in turn affect litter decomposition rates (Dearden & Wardle, 2008; He, Lin, Han, & Ma, 2013; Jackson, Nilsson, & Wardle, 2013; Yang, Wang, Huang, Hui, & Wen, 2014).

In wetland ecosystems, particularly in ponds and lakes, they are dominated and covered by free‐floating plants, for instance, *Lemna* in temperate regions (Pasztaleniec & Poniewozik, 2013; Scheffer et al., 2003) and *Eichhornia crassipes*, *Pistia stratiotes* and *Salvinia molesta* in tropical or subtropical regions (Attermeyer et al., 2016; Mbati & Neuenschwander, 2005; Wang et al., 2018). Free‐floating plants which are usually compact and formed by clonal growth can quickly spread and cover a large area of the water surface, especially as anthropogenic disturbances, temperature increases, and/or eutrophication are encountered (Mbati & Neuenschwander, 2005; Villamagna & Murphy, 2010; Wilson, Holst, & Rees, 2005). Such a compact canopy of free‐floating vegetation will retain leaf litter of riparian/lakeshore plants above the water surface (on free‐floating vegetation) for a period of time before sinking to the water bottom where decomposition is mainly completed in wetlands, such as lakes or ponds (Pintar & Resetarits, 2017; Rubbo et al., 2006; Stoler & Relyea, 2011, 2016; Wallace, Eggert, Meyer, & Webster, 1997; Webster & Meyer, 1997). Leaf litter that experiences a retention on free‐floating vegetation will be exposed to aerial conditions characterized by low water and nutrient availability (He et al., 2013), high temperature fluctuation (Kuehn, Churchill, & Suberkropp, 1998; Kuehn, Steiner, & Gessner, 2004), and low microbial productivity (Buesing & Gessner, 2006; Dearden & Wardle, 2008). In addition, such a retention may also reduce the chance and/or shorten the time that leaf litter is in contact with the benthic fauna that is among the major decomposers in wetlands, and this might cause a different subsidy for benthic fauna, which would have different roles on litter decomposition of later stage. Such dramatic differences in abiotic and biotic environments between the water surface and the water bottom might affect the decomposition rates of riparian leaf litter.

The decomposition process of leaf litter that experiences a retention period by free‐floating plants could be divided into two successive phases. The first phase will occur on the free‐floating vegetation. Free‐floating vegetation would intercept leaf litter as a barrier when leaf litter falls from riparian/lakeshore plants. However, those retained litter will eventually sink to the water bottom (the second phase) due to the movement by wind, water current, or human activities. This second phase in water bottom might be significantly altered by the presence of the first phase. For example, the retention of free‐floating plants might change the leaching, microbial conditioning, and fragmentation processes during above‐water‐surface decomposition compared to that happens in the water bottom (Aerts, 1997; Graça et al., 2015), which in turn will change the quality of litter which will be further processed by other decomposers, such as microbes or benthic fauna. However, few studies have tested the effects of such retention by free‐floating vegetation on litter decomposition process.

To explore how litter decomposes on the pond bottom after experiencing a retention on free‐floating vegetation, we conducted an in situ decomposition experiment of leaf litter from three native riparian species with contrasting life‐forms (and functional traits) in a wetland pond in the subtropical region of China. Leaf litter from the three species was incubated in litter bags with fine or coarse mesh sizes that remained on the bottom of the pond for 50 days or remained on free‐floating vegetation for 25 days before staying on the bottom of the pond for 25 days. We hypothesize that decomposition of the riparian leaf litter, that is, the losses of mass, carbon, and nitrogen, might be slowed down due to the retention of free‐floating plants, and such effects of free‐floating plant retention on leaf litter decomposition might depend on plant species identities which differ in their initial litter traits, and also depend on different groups of decomposers, notably the benthic fauna in the water. We predict the effects of free‐floating plant retention would be stronger on the species decomposing faster, due to its more available N to the decomposers in the water which might enlarge the differences between the retention and the control treatments.

## 2 MATERIALS AND METHODS

### 2.1 Study site and experimental materials

**Figure 1:**
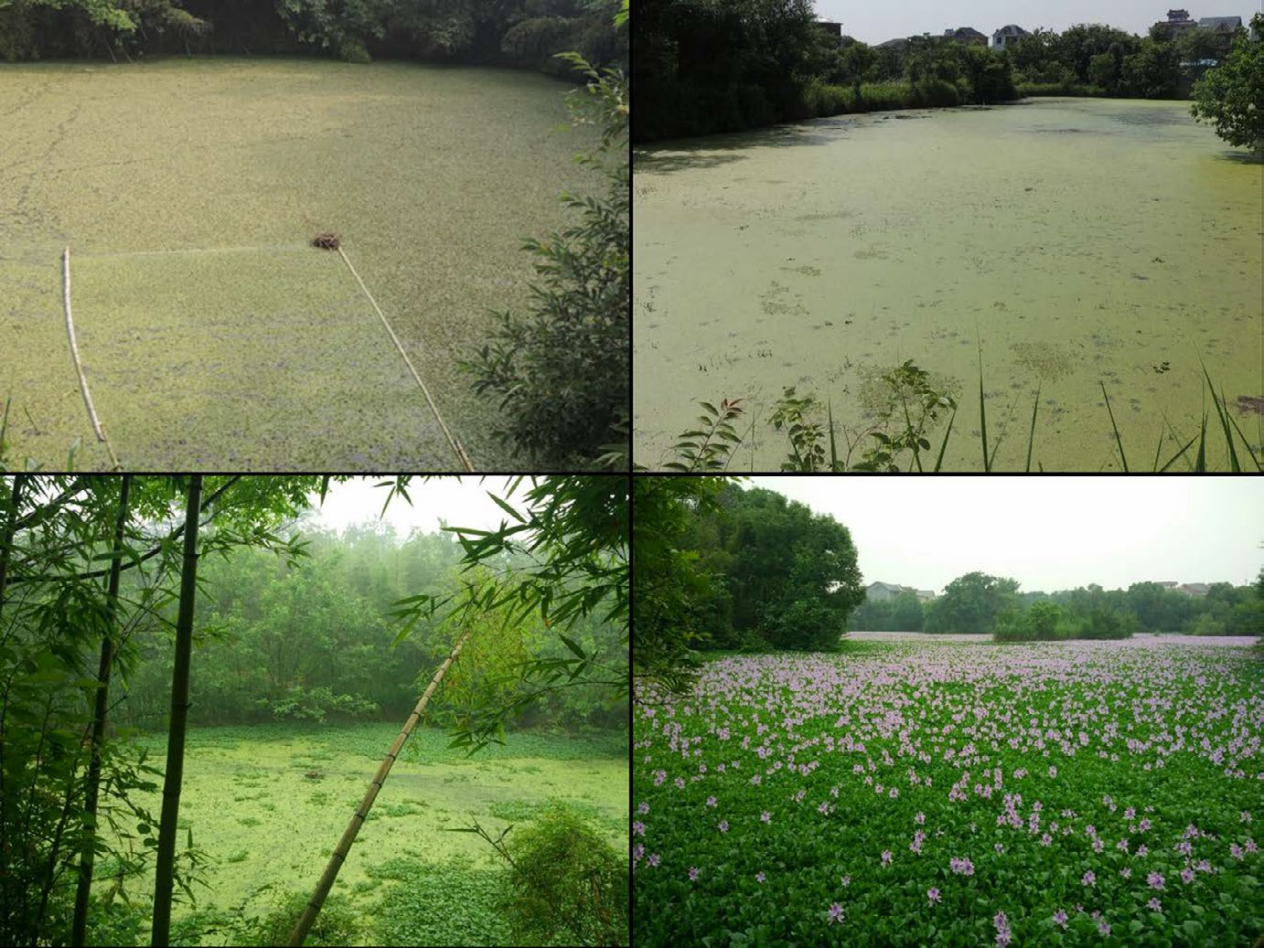
Some experimental ponds used in this study, which are covered by free‐floating vegetation

The experiment was performed in the Hemu Wetland (119°58′53″E–119°59′47″, 30°14′21″–30°15′08″N), which is located in the western part of Hangzhou City, Zhejiang Province, China. This area has a typical subtropical climate characterized by a hot and humid summer and a cold and dry winter, with a mean annual temperature of 17.5°C, a mean annual precipitation of 1,454 mm, and a relative humidity of 75%–85% (Zhang, Jiang, & Zhu, 2015). During the experiment, the mean air temperature was 33.5°C (range: 23–40°C), and rainy days accounted for 58% of the time (29/50 days). The wetland consists of hundreds of ponds with depths of 2–3 m. In many of the ponds, there are numerous compact vegetation patches that freely float on the water surface with varying sizes (Figure 1). Free‐floating vegetation is often dominated by clonal plants with strong clonal growth, such as *Azolla imbricata*, *Eichhornia crassipes*, *Lemna minor*, and *Salvinia molesta* (Zhou, 2015). Free‐floating vegetation can alter the conditions below and intercept the litter from riparian or emerged plants (Zhou, 2015). The retention of riparian plant litter on the floating vegetation before it sinks to the bottom of the ponds has also been observed in this area. The mean water temperature on the bottom was 26.7 ± 0.3°C, electrical conductivity was 217.50 ± 20.96 (μs/cm), pH was 6.96 ± 0.34, total nitrogen concentration was 0.93 ± 0.08 (mg/L), and total phosphorus concentration was 0.09 ± 0.08 (mg/L), respectively.

The leaf litter materials used for the experiment were from three riparian species with different life‐forms and leaf functional traits (Zhang et al., 2019), that is, an evergreen tree species (*Cinnamomum camphora* (L.) Presl.) with high leaf carbon content and low SLA, a deciduous tree species (*Diospyros kaki* Thunb.) with high leaf nitrogen content and low C/N ratio, and a perennial grass species (*Phyllostachys propinqua* McClure.) with high SLA, low thickness, and leaf carbon content. These species are dominant in the terrestrial habitats around the ponds, and their leaf litter often reaches the ponds and remains on the free‐floating vegetation before it sinks to the bottom of the pond (Zhang et al., 2019). Due to their phenological differences, freshly fallen leaves were collected from *D. kaki* in November 2015, *C. camphora* in April 2016, and *P. propinqua* in May 2016.

### 2.2 Experimental design

After the samples of leaf litters were air‐dried at room temperature in the laboratory, leaf litters of the same species were enclosed in the same nylon litter bags (15 × 20 cm) with mesh sizes of 0.5 mm (fine mesh size) and 2 mm (coarse mesh size), 4 ± 0.05 g litter per bag. The fine mesh size was used to exclude most macrofauna that may interfere with the microbial conditioning (Palmer, Straver, & Rundle, 2007).

There were two treatments in the experiment. In the first treatment (Treatment I), litter bags were placed on the free‐floating vegetation and stayed there for 25 days; then, the bags were moved to the pond bottom, and they stayed there for another 25 days, mimicking the decomposition of leaf litters on the pond bottom after they experience a retention on free‐floating vegetation for 25 days. In the second treatment (Treatment II), the litter bags were placed on the pond bottom and remained there for the entire experimental duration of 50 days, mimicking pond bottom decomposition of the leaf litters that do not experience a retention on free‐floating vegetation. A bottle of sand was attached to the same PVC tube device to force the leaf litter to sink to the bottom. All litter bags were randomly distributed in each pond with two treatments, and there were five ponds for each replication. The experiment lasted for 50 days from 13 June to 2 August 2016 during which the litter bags were retrieved twice. On the 25th day (8 July 2016) after the start of the experiment, we retrieved 15 litter bags per species per mesh size from the free‐floating vegetation in Treatment I and 15 litter bags per species per mesh size from the pond bottom in Treatment II. On the 50th day (2 August 2016, i.e., the final day) of the experiment, we retrieved 15 litter bags per species per mesh size from the pond bottom in both treatments I and II. In total, 120 litter bags were used: 2 treatments (retention or no retention on free‐floating vegetation before reaching the pond bottom) × 2 mesh sizes × 3 species × 2 harvest times × 5 replicates.

### 2.3 Measurements

In the laboratory, all leaf litter samples were gently and carefully washed with tap water so that the debris were removed from the leaf litters and then oven‐dried at 75°C for 48 hr before weighing. Mass loss was determined as the difference in dry weight before experiment and after each harvest time, and expressed as percentage of loss of dry weight to initial dry weight. Their total carbon and nitrogen concentrations were determined by an elemental analyzer (vario PYRO cube; Elementar), which expressed the concentrations as the percentage of dry mass (%).

### 2.4 Data analysis

Using the leaf litter dataset retrieved on 2 August 2016, that is, at the end of the experiment, three‐way ANOVAs were used to test for effects of the retention on free‐floating vegetation, species identity, mesh size, and their interactions on mass, carbon, and nitrogen losses from the leaf litter during decomposition, respectively. Duncan's test was performed if a significant difference was detected.

Using the leaf litter dataset retrieved on 8 July 2016, three‐way ANOVAs were applied to test for effects of decomposition condition (i.e., on free‐floating vegetation vs. on pond bottom), species identity, mesh size, and their interactions on mass, carbon, and nitrogen losses in the first half (25 days) of the experiment, respectively. Duncan's test was performed if a significant difference was detected. Normal distribution and homogeneity of variances were checked by Shapiro–Wilk's and Levene's test, respectively, before ANOVA, and all data were normally distributed and met the assumption of homogeneity of variance. All analyses were conducted with SPSS 20.0 software (IBM SPSS Statistics).

## 3 RESULTS

### 3.1 Main effects of three factors on litter decomposition

There were (marginally) significant negative effects of the retention of free‐floating vegetation on litter mass and carbon losses after both harvests times (Table 1, *p* = .059 and *p* < .001 for 50 days and first 25 days, respectively), but there was no effect of such retention on litter nitrogen losses (Table 1). For the species identity, there was significant effect of species identity on litter mass, carbon, and nitrogen losses after both harvests (Table 1), and the decomposition rate was the highest for *P. propinqua*, followed by *D. kaki* and *C. camphora*. Moreover, there was also significant effect of mesh size on the litter mass loss after both harvests times (Table 1, *p* < .001 and *p* = .028 for 50 days and first 25 days, respectively), but significant effect of mesh size on litter carbon and nitrogen loss was only observed in 50 days harvest time (Table 1).

**Table 1:** Results of three‐way ANOVA used to test for the effects of retention on free‐floating vegetation (V), species (S), mesh size (M), and their interactions (V × S, V × M, M × S, and V × M × S) on mass loss (M loss), carbon loss (C loss), and nitrogen loss (N loss) from riparian leaf litter during 25 and 50 days of decomposition

Factor	V	S	M	V × S	V × M	M × S	V × M × S
Decomposition for 50 days							
M loss							
*df*	1.48	2.48	1.48	1.48	2.48	2.48	2.48
*F*	3.74	9.76	11.80	5.56	0.17	1.86	1.42
*p*	*.059*	***<.001***	***.001***	***.007***	.686	.167	.252
Explained variation %	3.70	19.34	11.70	11.03	0.16	3.68	2.81
C loss							
*df*	1.48	2.48	1.48	1.48	2.48	2.48	2.48
*F*	10.05	24.96	5.66	7.64	0.51	1.51	1.01
*p*	***.003***	***<.001***	***.021***	***.001***	.480	.231	.372
Explained variation %	7.48	37.12	4.21	11.37	0.38	2.25	1.50
N loss							
*df*	1.48	2.48	1.48	1.48	2.48	2.48	2.48
*F*	1.69	59.97	4.63	0.06	0.564	11.36	1.46
*p*	.200	***<.001***	***.037***	.937	.456	***<.001***	.243
Explained variation %	0.84	59.80	2.31	0.064	0.28	11.32	1.46
Decomposition for the first 25 days							
M loss							
*df*	1.48	2.48	1.48	1.48	2.48	2.48	2.48
*F*	15.18	9.11	5.14	8.45	0.46	0.49	1.50
*p*	***<.001***	***<.001***	***.028***	***.001***	.501	.615	.233
Explained variation%	14.07	16.89	4.76	15.65	0.42	0.91	2.78
C loss							
*df*	1.48	2.48	1.48	1.48	2.48	2.48	2.48
*F*	3.92	25.45	0.45	12.06	0.12	0.88	0.19
*p*	*.053*	***<.001***	.505	***<.001***	.726	.420	.829
Explained variation %	3.03	39.25	0.35	18.60	0.095	1.36	0.29
N loss							
*df*	1.48	2.48	1.48	1.48	2.48	2.48	2.48
*F*	1.57	68.74	1.52	11.80	2.73	0.74	1.75
*p*	.216	***<.001***	.224	***<.001***	.105	.481	.186
Explained variation %	0.72	62.52	0.69	10.74	1.24	0.68	1.59

Note

The *p* values <.05 are in bold and italics and indicate significance at *p* = .05.

### 3.2 Interactive effects among three factors on litter decomposition

**Figure 2:**
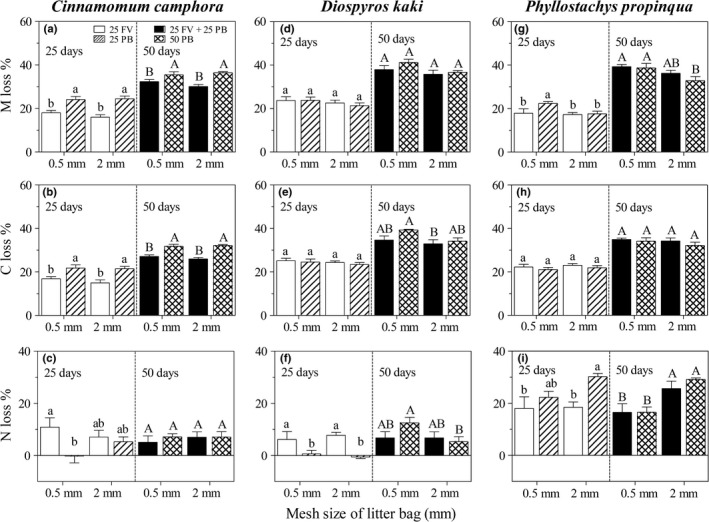
Mass loss (M loss), carbon loss (C loss), and nitrogen loss (N loss) from leaf litters in litter bags with mesh sizes of 0.5 or 2.0 mm, and each incubated for 25 days (25PB) or 50 days on the pond bottom (50PB), 25 days on free‐floating vegetation (25FV), or 25 days on free‐floating vegetation before another 25 days on the pond bottom (25FV + 25PB). Panels (A), (B), and (C) are for *Cinnamomum camphora*, (D), (E), and (F) are for *Diospyros kaki*, and (G), (H), and (I) are for *Phyllostachys propinqua*. In the same subpanel, the bars sharing the same letter are not different at *p* = .05

There was significant interactive effect between the retention of free‐floating vegetation and the species identity on litter mass and carbon losses in both harvest times, but this interactive effect on nitrogen loss was only observed in first 25 days harvest time (Table 1; Figure 2; *p* < .001). Moreover, there was a significant interactive effect of the species identity and mesh size on litter nitrogen loss in 50 days harvest time (Table 1; Figure 2).

Specifically, after the first 25 days, retention on free‐floating vegetation significantly decreased the mass and carbon losses from *C. camphora* litter for both mesh sizes, but significantly increased the nitrogen loss from *C. camphora* litter in small mesh size (Figure 2); retention effect was found only for nitrogen loss from *D. kaki* litter (Figure 2); and retention on free‐floating vegetation significantly increased the mass loss from *P. propinqua* litter in small mesh size, and also increased the nitrogen loss in large mesh size (Figure 2). Moreover, after 50 days, retention on free‐floating vegetation significantly decreased the mass and carbon losses from *C. camphora* litter but did not decrease the losses from *D. kaki* or *P. propinqua* litters (Figure 2).

## 4 DISCUSSION

Our study found that a 25‐day retention of riparian leaf litter on free‐floating vegetation significantly affected the carbon loss and marginally affected the mass loss throughout the 50‐day experimental period. Meanwhile, the interactive effects between the retention and species identity on mass and carbon losses were significant. Additionally, during the first 25 days of the experiment, both mass and carbon losses from the leaf litter that decomposed on free‐floating vegetation were lower compared to the plant litter that decomposed on the pond bottom. Meanwhile, such effects were species‐dependent. These findings suggest that the retention of riparian leaf litter on free‐floating vegetation affects the subsequent decomposition on the bottom. To the best of our knowledge, this is the first study that reveals how the decomposition of plant litter in wetland ecosystems is affected by a retention on free‐floating vegetation.

**Figure 3:**
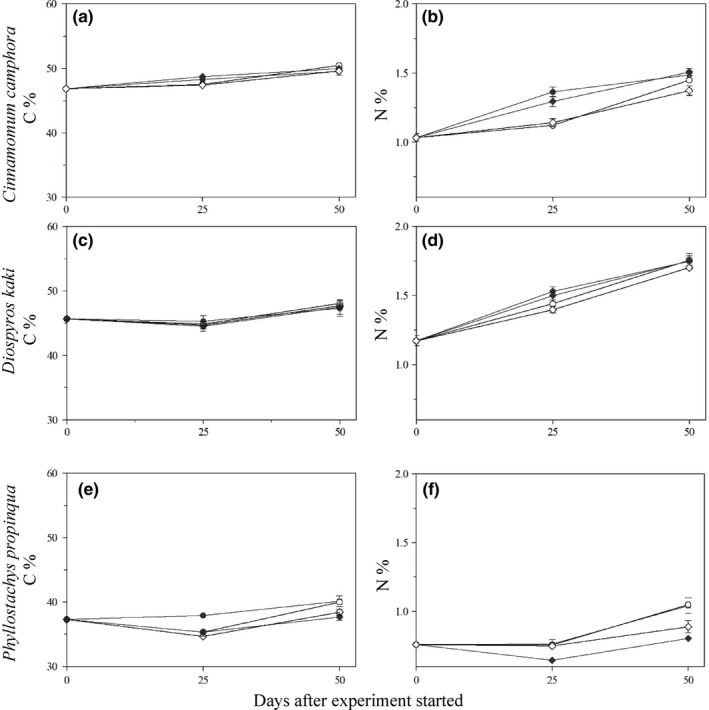
Changes in the total carbon concentration (C %) and nitrogen concentration (N %) of leaf litter during the experiment. Panels (A) and (B) are for *Cinnamomum camphora*, (C) and (D) are for *Diospyros kaki*, and (E) and (F) are for *Phyllostachys propinqua*. Solid circles, solid diamonds, open circles, and open diamonds stand for the litter bags with mesh sizes of 0.5 and 2.0 mm on the pond bottom and litter bags with mesh sizes of 0.5 and 2.0 mm on the pond bottom after retention on floating vegetation, respectively

As we predicted, the decomposition of *C. camphora* leaf litter showed a significant decrease in mass loss in response to the interception by free‐floating vegetation, which is also consistent with the results of previous studies in terrestrial ecosystems that found similar decreases when leaf litters were intercepted by understory vegetation and attributed them to inhibition of microbial activities (biotic factors) via water and nutrient limitations (abiotic factors; He et al., 2013; Yang et al., 2014). However, we did not observe an effect of the retention on free‐floating vegetation for *D. kaki* and *P. propinqua*. These findings imply that the retention effect in wetland ecosystems depends on species identity and/or leaf functional traits. The possible reason for this difference might be that these species contain some particular substances that are less palatable to decomposers. Evergreen tree species generally show a decomposition rate that is lower than that of deciduous tree species, resulting from their low palatability due to high C/N ratio or lignin contents (Decker & Boerner, 2006; Liu et al., 2016). Although *D. kaki* and *C. camphora* litters have similar initial carbon (45.67 ± 0.25 and 46.86 ± 0.26 g/g, respectively) and nitrogen concentrations (1.17 ± 0.04 and 1.03 ± 0.03 g/g, respectively) and similar nitrogen dynamics, their carbon dynamics were different in the first stage of this study (Figure 3). The increased carbon content in the *C. camphora* litter in the first stage might indicate the relatively persistent recalcitrant carbon compounds in its substrate (Berg & McClaugherty, 2014). Available carbon and nitrogen are the main requirements for the microbial growth (Pastor et al., 2014). Therefore, the decomposition environment on free‐floating vegetation might not be conducive to the decomposition of *C. camphora* litter, resulting in the more sensitive response than of *D. kaki*. In addition, the nitrogen dynamics of *P. propinqua* were different from those of *C. camphora* and *D. kaki*. For *P. propinqua*, the nitrogen content changed little, and the nitrogen losses were almost the same between the litter on free‐floating vegetation and that in the first phase of the experiment. This result indicates that bamboo leaf litters might resist microbial colonization due to silica deposition, as reported by Motomura, Fujii, and Suzuki (2004). Previous study also found Poales species (e.g., *P. propinqua* in this study) usually have higher silica content than other monocot clades (Hodson, White, Mead, & Broadley, 2005). The possible reason for this difference might be that silica content could inhibit the fungus growth during decomposition (Schaller, Hines, Brackhage, Bäucker, & Gessner, 2016; Xiong et al., 2017). No effect of the retention was detected in *P. propinqua* litter. This result may be due to the immobilization of dominant bacteria colonization resulting from a partial solution of phytoliths and amorphous silica in the latter stages after the leaf material was partially broken down (Gessner, Chauvet, & Baldy, 1995; Schaller et al., 2016; Wright & Covich, 2005).

Our study showed that a retention on free‐floating vegetation seemed to have a significant but limited impact on litter decomposition (4.72%) in the wetland ecosystem at least in a shorter timescale. First, this result could be partly due to the relatively short period of time (25 days) in which the litter remained on free‐floating vegetation in the present study. He et al. (2013) revealed that the retention (i.e., interception in their terms) effect would increase with an increase in retention durations such as 120 days in their study. Dearden and Wardle (2008) found that litter interception increased litter decomposition by from 0.43% for *Weinmannia racemosa* to 12.15% for *Nothofagus menziesii* after 360 days of incubation. Second, this result could be due to the high frequency (68%) of rainfall events (17/25 days) during the retention. It has been reported that microbial respiration would increase when exposed to wetting conditions (Kuehn & Suberkropp, 1998). Thus, this condition might narrow the differences in the rates of litter decomposition between those on free‐floating vegetation and those on the pond bottom, and these differences are driven by the contrasting incubation environments.

In this study, no significant difference in mass loss was detected between the two mesh sizes in both free‐floating vegetation and on the pond bottom, suggesting that microorganisms predominantly controlled litter decomposition. Therefore, the magnitude of the variance caused by the retention will not be affected by mesh size. In fact, in the first phase of the experiment, we found some fauna in the litter bags with coarse mesh (2 mm) on the free‐floating vegetation, such as spiders and ants. However, nonsignificant impact of mesh size was detected on the free‐floating vegetation, indicating its limited influence on litter decomposition. Ants and spiders have trophic cascade effects on the detritus‐based food web. However, their effects on litter decomposition are inconsistent (Ii & Bradford, 2012; Lawrence & Wise, 2000, 2004; Mcglynn & Poirson, 2012), which depends on the extent of the trophic cascade effect and the interaction between microorganisms and fauna (Miyashita & Niwa, 2006). In our study, the proportions of litter bags with spiders and ants were relatively low (8.9%), which might result in a weak trophic cascade effect. The nonsignificant effect of mesh size on litter decomposition on the pond bottom suggests that macroinvertebrates might have little effect on litter decomposition in our experimental system, and we only found benthic fauna existed in one of the coarse mesh bag. Other potential explanation might be there is lack of macroinvertebrates in the wetland we studied, because this wetland suffered heavily human disturbances (Dong et al., 2013).

In fact, the period when the litter remained on the understory vegetation also depended on the morphology and structure of the understory vegetation (Alvarez‐Sanchez & Guevara, 1999). Some free‐floating plants (such as *E. crassipes* and *P. stratiotes*) have broad, water‐resistant leaves and extensive branching roots. Other species, such as Lemnaceae, Azollaceae, and Salviniaceae, have small leaves and degraded root systems (Boutin & Keddy, 1993). Thus, the different species that constitute free‐floating vegetation might serve different functions that would affect the time of plant litter retention on the vegetation. Therefore, we suggest that more types of free‐floating vegetation and more durations of leaf litter retention should be considered in future research. Furthermore, except the retention effect tested in the current study, the free‐floating vegetation can also form physical coverage water surface, which could modify the biotic and abiotic factors in pond bottom, and consequently change ecosystem processes such as litter decomposition. Thus, the effects of free‐floating vegetation could be understood from the perspective of biotic and abiotic factors they modified.

## 5 CONCLUSIONS

Our in situ experiment found that the retention of (allochthonous) riparian leaf litters on free‐floating vegetation had significant negative effects on carbon losses, marginal negative effects on mass losses, and no effect on nitrogen losses, indicating that free‐floating vegetation could play a certain extent role in regulating the carbon cycle in wetland ecosystems such as the ponds in our study. Similarly, mass and carbon losses from riparian leaf litter that decomposed on a pond bottom for the first 25 days of the experiment were greater than those from the litter that decomposed on free‐floating vegetation. We suggest that more studies should be conducted in the future to provide a deeper understanding of the ecological role of free‐floating vegetation in litter decomposition, which is linked to the nutrient cycles of wetland ecosystems.

## CONFLICT OF INTEREST

None declared.

## AUTHOR CONTRIBUTIONS

MD and YBS designed the experiment. YLZ, WJZ, and JPD performed the experiment. YLZ, XP, and YBS analyzed the data. All authors contributed critically to the drafts and gave final approval for publication.

## Data Availability

The data associated with this manuscript have been deposited in Dryad with https://doi.org/10.5061/dryad.392gf43.
